# Label-Free Glucose Detection Using Cantilever Sensor Technology Based on Gravimetric Detection Principles

**DOI:** 10.1155/2013/687265

**Published:** 2013-08-01

**Authors:** Shuchen Hsieh, Shu-Ling Hsieh, Chiung-wen Hsieh, Po-Chiao Lin, Chun-Hsin Wu

**Affiliations:** ^1^Deparment of Chemistry, National Sun Yat-Sen University, 70 Lien-Hai Road, Kaohsiung 80424, Taiwan; ^2^Department of Seafood Science, National Kaohsiung Marine University, Kaohsiung 81157, Taiwan; ^3^Department of Computer Science and Information Engineering, National University of Kaohsiung, Kaohsiung 81148, Taiwan

## Abstract

Efficient maintenance of glucose homeostasis is a major challenge in diabetes therapy, where accurate and reliable glucose level detection is required. Though several methods are currently used, these suffer from impaired response and often unpredictable drift, making them unsuitable for long-term therapeutic practice. In this study, we demonstrate a method that uses a functionalized atomic force microscope (AFM) cantilever as the sensor for reliable glucose detection with sufficient sensitivity and selectivity for clinical use. We first modified the AFM tip with aminopropylsilatrane (APS) and then adsorbed glucose-specific lectin concanavalin A (Con A) onto the surface. The Con A/APS-modified probes were then used to detect glucose by monitoring shifts in the cantilever resonance frequency. To confirm the molecule-specific interaction, AFM topographical images were acquired of identically treated silicon substrates which indicated a specific attachment for glucose-Con A and not for galactose-Con A. These results demonstrate that by monitoring the frequency shift of the AFM cantilever, this sensing system can detect the interaction between Con A and glucose, one of the biomolecule recognition processes, and may assist in the detection and mass quantification of glucose for clinical applications with very high sensitivity.

## 1. Introduction

Diabetes mellitus (DM) is a serious public health concern that causes illness, disability, and death throughout the world. Clinical diabetes therapy requires precise monitoring and maintaining of blood glucose levels as close to normal as possible to reduce the risk of emergency complications such as the retinopathy, nephropathy, and hypo- and hyperglycemia [1–3]. To date, existing sensing techniques have been demonstrated, such as glucose-based noninvasive glucose sensors and enzyme-based biosensors; however, impaired responses and unpredictable drift make these unsuitable for long-term therapeutic practice [3]. Therefore, the development of new diagnostic tools for reliable glycemic control with sufficient sensitivity and selectivity is of clinical interest.

In order to solve these problems, microcantilever-based sensors have been proposed, which have high sensitivity, low detection limits, and broad application in the fields of chemistry, biotechnology, detection, and medical science [4, 5]. This sensing system has significant advantages over other sensor technologies, such as high surface-to-volume ratio, fast response time, and low cost of fabrication [4], and further, has proven effective in DNA hybridization and, biomolecular recognition [6]. Moreover, attogram-level mass detection sensitivities have been achieved using cantilever-based sensing technology [7]. 

Microcantilevers have been used as force probes in atomic force microscopy (AFM) and for high-resolution imaging to examine the topography and mechanical properties of biological samples without labels or treatment. Further, the cantilever may be functionalized with various probe molecules (e.g., DNA, proteins, and antibodies) and used as a specific biosensor for target molecules (e.g., DNA and antigens) [8]. This sensing system can be further specialized to act as a functional biosensor, whereby the resonance frequency of the cantilever shifts with mass loading when biomolecules absorb onto a treated tip [5]. 

In the present study, we have used microcantilevers (via the vibration method) and measured the resonance frequency shifts for glucose recognition and mass detection. We chemically modified the silicon AFM microcantilevers with aminopropylsilatrane (APS), followed by adsorption of concanavalin A (Con A) onto the APS-modified probe. Glucose-specific lectin Con A has a strong affinity for glucose and has been used as a glucose monitoring sensor [9, 10]. We used the Con A/APS-modified probe to detect glucose and galactose in solution and then compared the frequency shifts to determine which type of sugar attached to the Con A. We demonstrate that the AFM-based microcantilever method of glucose detection offers a powerful technology which can achieve attogram mass sensitivity in clinical microbiology systems for management of glucose levels of DM patients. 

## 2. Materials and Methods

### 2.1. Sample Preparation

An aminopropylsilatrane (APS) solution was prepared using a synthetic method reported earlier [11, 12], and a stock sample of 50 mM APS in Milli-Q reagent grade (type I) water (18.2 MΩ-cm at 25°C) was prepared and stored at 4°C. [13]. Con A, from *Canavalia ensiformis* (Jack bean) (type VI, lyophilized power, MW 26.5 kDa), was purchased from Sigma-Aldrich (USA) and was used to prepare a 1000 ppm Con A solution. D(+) glucose (dextrose anhydrous) was purchased from SHOWA, and was used to prepare a 1% glucose solution. D(+) galactose was purchased from Sigma-Aldrich (USA) and was used to prepare a 1% galactose solution. Throughout the experiments, Milli-Q reagent grade (type I) water (18.2 MΩ-cm at 25°C) was used.

### 2.2. Ultrasensitive Functionalized Probe for Glucose Detection

All cantilever probes were plasma cleaned [14] (Harrick Scientific Products, Inc.) for 2 min using dry air as the reactive gas to increase the OH concentration at the surface [15, 16]. This process creates a uniformly hydroxylated surface. Arrow force modulation mode probes (Arrow-FMR) were purchased from NanoWorld AG (Switzerland). The cantilevers were 240 *μ*m long, 35 *μ*m wide, had a triangular free end, and tetrahedral tip shape with a height of 10–15 *μ*m with a measured normal spring constant of 2.8 N/m (thermal method) [17, 18].

To prepare the probes for glucose detection, plasma cleaned cantilevers were immersed in a diluted APS solution (volume ratio of APS : water = 1 : 300) for 30 min, removed, rinsed thoroughly with milli-Q water, and then dried under a stream of pure nitrogen gas. Following this procedure, alkylsilane molecules were chemically attached to the AFM tip, much like self-assembled monolayers on silicon substrates from our previous study [13]. We then put the APS-coated cantilever into a 1000 ppm Con A solution for 15 min, removed and rinsed thoroughly with milli-Q water, and then dried under a stream of pure nitrogen gas. Finally, the APS/Con A modify cantilever was immersed in a 1% glucose solution for 15 min, rinsed thoroughly with milli-Q water, and dried under a stream of pure nitrogen gas.

Sections of a silicon (100) wafer (TSR Technology Inc.; P-type/boron dopant; resistivity 1–10 Ω^3^ cm) were used for complimentary experiments where the Si substrates were treated with APS and Con A and then exposed to glucose or galactose. Changes in surface roughness and topography, as measured by AFM, were related to glucose or galactose adsorption. 

**Figure 1:**
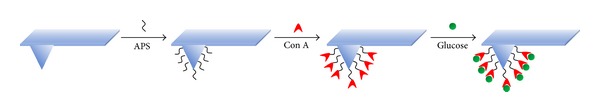
Schematic drawing of the cantilever modification procedure glucose detection by attachment.

Topographic images were obtained using an AFM (MFP-3D, Asylum Research, Santa Barbara, CA, USA) operating in AC mode under ambient conditions. A silicon cantilever (Nano World AG, Arrow-FMR) with a nominal spring constant of 2.8 N/m was used for all images, with a scan rate of 1.0 Hz and image resolution of 512 × 512 pixels.  Figure 1 is a cartoon depicting the process of chemically functional AFM tip to attach the sample.

## 3. Results and Discussion

### 3.1. Resonance-Frequency-Based Gravimetric Biosensor

**Figure 2:**
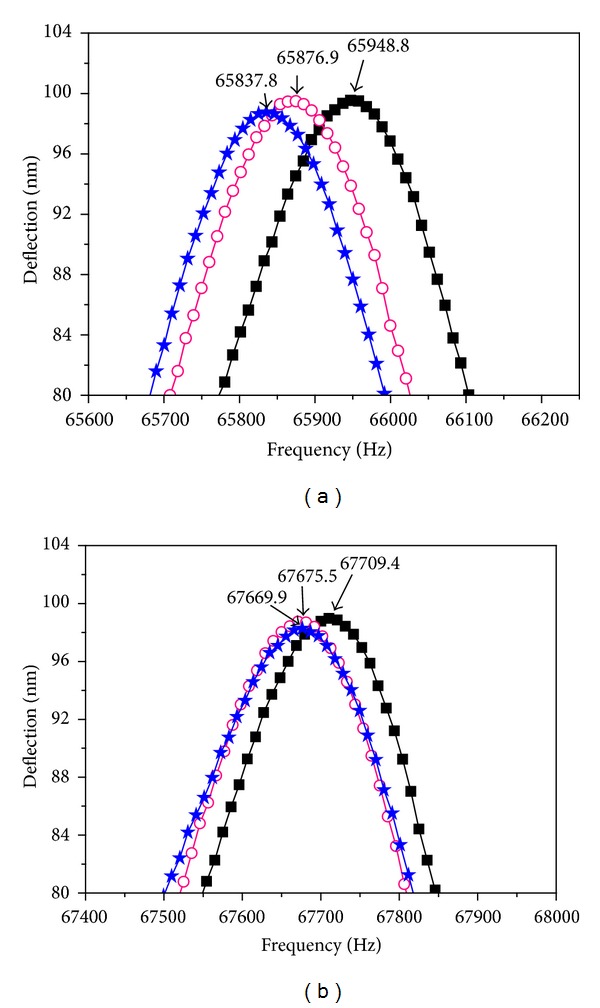
Cantilever frequency shifts at each step in the cantilever modification process for (a) glucose and (b) galactose. The symbols (■, ○, and ★) represent APS, APS/Con A, and APS/Con A/glucose or galactose, respectively.

As shown in Figure 2(a) (■), the APS-modified cantilever had a resonance frequency of 65,948.8 ± 5.0 Hz in air. Con A was chemically attached to the cantilever by immersing the cantilever into the Con A (1000 ppm) solution for 15 mins and then immersing the probe into ultrapure water to rinse off the unbonded residues. Figure 2(a) (○) shows that the resonance frequency after the addition of Con A was 65,876.9 ± 5.5 Hz. The resonance frequency of the cantilever is given by [19]

(1) $$\begin{array}{c} f=\frac{1}{2\pi }\sqrt{\frac{k}{M}}, \end{array}$$

where *k* is the spring constant and *M* is the effective mass of a cantilever. The measured downshift in the cantilever resonance frequency confirms that the Con A molecules have attached to the tip, and these molecules have increased the cantilever effective mass. The frequency shift obtained using (1) was used to estimate an effective mass increase of 9.13 pg, which corresponds to 3.45 × 10^−16^ moles of Con A attached to the cantilever. 

The same probe was then used to detect glucose by incubating the probe in the prepared glucose solution and then rinsing in ultrapure water to remove the unbonded residues and drying. Immediately following glucose exposure, we observed that the cantilever resonance frequency had shifted lower to 65,837.8 ± 2.8 Hz as shown in Figure 2(a) (★). Using the same calculation, we estimated an effective mass increase of about 4.98 pg, which corresponds to the addition of 2.76 × 10^−14^ moles of glucose to the probe.

As a negative control, we ran a similar experiment using galactose rather than glucose as the analyte. Although the molecular formulas for glucose and galactose are identical, they are structural isomers, where the difference is the turning up (or down) of the hydroxyl group on one of the carbons. Using the same process, a coated Con A-modified probe was immersed into the galactose solution for 10 min and then immersed in ultrapure water to rinse off the unbonded residues. As shown in Figure 2(b) (■), the APS-modified cantilever exhibited a resonance frequency of 67,709.4 ± 6.0 Hz. Following Con A attachment, the resonance frequency shifted to 67675.5 ± 3.5 Hz (○), which corresponded to a mass increase of 4.6 pg, or 1.73 × 10^−16^ moles of Con A attached to the cantilever. After incubation in the galactose solution, the measured resonance frequency was 67,669.9 ± 3.0 Hz (★) which was within the measurement error of the Con A (only) measurement (○) and indicates that galactose did not attach to the Con A-modified AFM probe.

### 3.2. Measuring Surface Roughness with AFM Imaging

**Figure 3:**
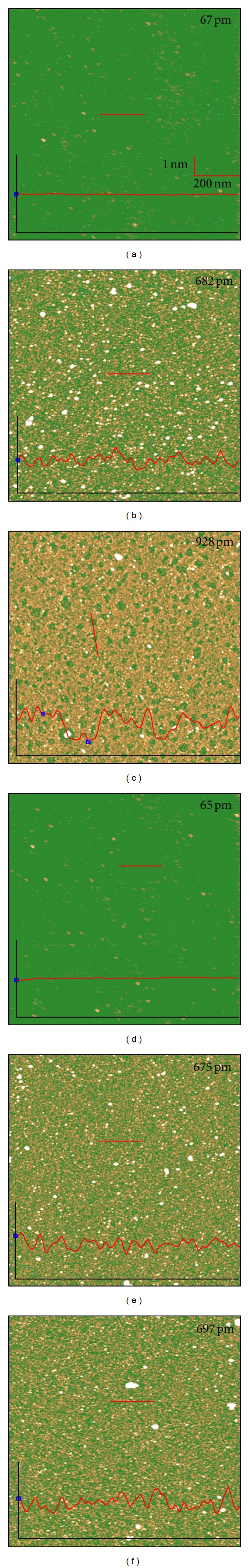
Topographic images (height data) (a, d) of an APS-modified silicon surface. Topographic images (b, e) of a Con A/APS/Si wafer. Upper right hand corner shows the surface roughness of the scanned area. Images (c) and (f) show the Con A/APS/Si wafer after exposure to glucose and galactose, respectively. The glucose-exposed surface exhibits a significantly modified topography, while no change is observed for the sample exposed to galactose. All images are 5 × 5 *μ*m^2^.

To verify the detection step of our method, we ran a similar experiment on a silicon wafer substrate and monitored morphology changes using AFM topographical imaging. The results are shown in Figure 3. The same sample preparation procedures were used for the silicon wafer substrates as were used for the silicon microcantilevers. Figures 3(a) and 3(d) show AFM images of the APS-modified surface morphology. The surfaces were very flat, with root mean square (RMS) roughness of 67 and 65 pm, respectively. Figures 3(b) and 3(e) show the same substrates after Con A was adsorbed onto the APS-modified surfaces. The surface roughness increased for both surfaces to 682 and 675 pm, respectively. The surfaces shown in Figures 3(b) and 3(e) were then treated with glucose and galactose, respectively. The surface treated with glucose (Figure 3(c)) exhibited an increase in RMS roughness to 928 pm and evolved a stepped topography with a network of holes ~1.5 nm deep. The surface shown in Figure 3(f), on the other hand, showed similar topography as that for the Con A-modified surface (Figure 3(e)) with a negligible change in surface roughness (697 pm). 

Within each of the Figure 3 topography images, we plotted line scan profiles to more clearly display the surface topography. The straight red line in each image shows where the cross-section data was obtained. In Figures 3(a) and 3(d), the line scan is flat, indicating that the surface is very smooth. Figures 3(b) and 3(e) show that the line section over a 1 *μ*m range has a peak to valley height of 650 pm. The line section in Figure 3(c) shows that the holes have a depth ~1.5 nm. All the line sections are plotted on the same scale, with the scale bar shown in Figure 3(a). These results indicate that glucose adsorbed onto the Con A-treated surface, while galactose did not. This further confirms the results from our cantilever resonance frequency measurements where glucose (but not galactose) was detected.

## 4. Conclusions

We have demonstrated a simple and effective method for detecting glucose using a chemically modified AFM probe and estimating the change in mass by monitoring the resonance frequency shift of the cantilever. This method may be used to investigate lectin-carbohydrate interactions in the form of carbohydrate and lectin arrays or more broadly to study a wide range of cell-cell and antigen-antibody bonds. With attogram mass sensitivity, this technique shows great promise for the development of frequency detection sensor technology.
